# Ultrasound radiomics features predicting the dosimetry for focused ultrasound surgery of benign breast tumor: A retrospective study

**DOI:** 10.3389/fgene.2022.969409

**Published:** 2022-09-06

**Authors:** Mengdi Liang, Cai Zhang, Tiansong Xia, Rui Chen, Xinyang Wang, Miaomiao Weng, Hui Xie, Lin Chen, Xiaoan Liu, Shui Wang

**Affiliations:** ^1^ Department of Breast Surgery, The First Affiliated Hospital with Nanjing Medical University, Nanjing, China; ^2^ State Key Laboratory of Ultrasound in Medicine and Engineering, Chongqing Medical University, Chongqing, China; ^3^ Chongqing Key Laboratory of Biomedical Engineering, Chongqing Medical University, Chongqing, China

**Keywords:** focused ultrasound surgery (FUS), benign breast tumor, sonication energy, dosage delivery, radiomics analysis

## Abstract

**Purpose:** To investigate the correlation between pre-ablation ultrasound radiomics features and the sonication energy for focused ultrasound surgery (FUS) of benign breast tumors.

**Method:** 53 benign breast tumors of 28 patients treated by ultrasound-guided focused ultrasound surgery (USgFUS) were included in this study. The sonication energy per unit volume of each tumor was calculated. Three-quarter point was chosen as the cut-off to divide the 53 included tumors into high sonication energy (HSE, *n* = 14) and low sonication energy (LSE, *n* = 39) groups. For each tumor, the region of interest (ROI) of both the tumor itself (tROI) and the near field tissue (nfROI) were delineated and analyzed separately using ImageJ software. Pearson correlation coefficient and multiple linear regression analysis were used for radiomics feature selection. To explore the diagnostic performance of different ultrasound radiomics features, a receiver operating characteristic (ROC) curve analysis was performed.

**Results:** In total of 68 radiomics features were extracted from pre-ablation ultrasound images of each tumor. Of all radiomics features, BX in tROI (*p* < 0.001), BX (*p* = 0.001) and Circ (*p* = 0.019) in nfROI were independently predictive features of sonication energy per unit volume. The ROC curves showed that the area under the curve (AUC) values of BX in tROI, BX, and Circ in nfROI were 0.797, 0.787 and 0.822, respectively.

**Conclusion:** This study provided three radiomics features of pre-ablation ultrasound image as predictors of sonication dose for FUS in benign breast tumors. Further clinical trials are needed to confirm the predictive effect of these radiomics features.

## Introduction

Benign breast tumors are the most common complaints in females and attack more frequently than malignant ones do. Fibroadenoma, which consists of both fibrous and glandular tissue, is the most common benign tumor in the female breast (Dent and Cant, 1989). It occurs at any age in the reproductive period. And hormone-related changes can induce a slight increase in size during pregnancy (Greenberg et al., 1998).

The majority of patients presenting with breast masses choose surgery resection rather than serial observation because of bothersome prominence, intermittent growth, physical discomfort, and anxiety (Kaufman et al., 2002; Kaufman et al., 2004; Yu et al., 2017). Common methods for tumor resection are conventional surgery, vacuum or endoscopy-assisted minimal invasive surgery (Yom et al., 2009; Lai et al., 2017). However, an unsightly scar and unnecessary excision of normal tissue often result in concerns about cosmesis defects and effects on breastfeeding. The advent of ablation techniques provides an office-based minimal invasive treatment which may reduce discomfort, shorten healing time and has limited scarring (Hynynen et al., 2001; Kaufman et al., 2002; Kaufman et al., 2004; Littrup et al., 2005; Dowlatshahi et al., 2010; Teh and Tan, 2010; Li et al., 2016; Yu et al., 2017; Zhou et al., 2017; Li et al., 2018).

Focused ultrasound surgery (FUS) is the only noninvasive transcutaneous ablative therapy which converges multiple beams of high-intensity ultrasound in the target area. The energy within the area is sufficient to induce irreversible cell damage, protein denaturation, and coagulative necrosis (Izadifar et al., 2020). FUS has been effectively used in treating various kinds of solid tumors (Izadifar et al., 2020), besides benign and malignant breast tumors (Hynynen et al., 2001; Wu et al., 2005; Cavallo Marincola et al., 2015; Kovatcheva et al., 2017; Imankulov et al., 2018; Peek et al., 2018; Kwong et al., 2021). Although FUS has been repeatedly shown to be feasible and promising, its widespread acceptance has been limited because of the relatively long ablation time and low complete ablation rate (Zhou et al., 2012; Peek et al., 2015; Peek et al., 2016; Peek et al., 2017). It is partially due to some technical factors (Hynynen et al., 2001), such as the difficulty of controlling focal spot position, precise target definition, and beam dosimetry. Besides technical factors, body tissue would play a determining role in dose-effect relation to FUS treatment. Previous studies showed that the biological focal region (BFR) of FUS differed in various tissue structures (Wang et al., 2003). Our clinical experience has indicated a small number of benign breast tumors require folds of energy to be ablated completely. The significant increase in the sonication dose may lead to prolonged treatment duration, increased incidence of complications, and incompleted ablation. It would be useful to determine in advance which kind of tumors need much more energy by FUS and which not.

Ultrasonography, including greyscale ultrasonography, color Doppler ultrasonography, ultrasound elastography and contrast-enhanced ultrasound, is the primary modality utilized for evaluation of breast masses. Radiomics can provide a large amount of high-dimensional quantitative image features (termed radiographic phenotypes) from medical images, which has the potential to identify features or combinations of features among patients with similar conditions and predict outcomes (Gillies et al., 2016; Yang et al., 2020). Tumors were ablated *in situ* by FUS and were not removed, so detailed histologic information was not available. Therefore, it is of great significance to fully excavate the ultrasound image features. And radiomics is expected to reveal ultrasound image characteristics of lesions related to different therapeutic effects. This study aimed to investigate the correlation between pre-ablation ultrasound radiomics features and different levels of the sonication energy for FUS of benign breast tumors.

## Materials and methods

### Patients with focused ultrasound surgery

**FIGURE 1:**
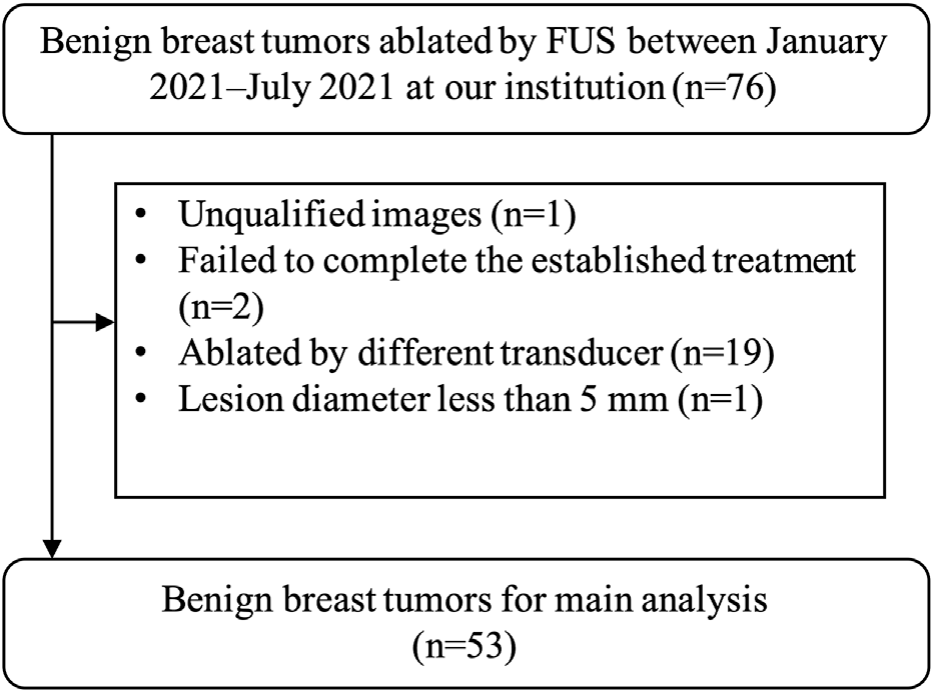
The results of the patients’ enrolments. In total, 53 tumors of 28 patients were enrolled in this study. FUS, focused ultrasound surgery.

This retrospective study of imaging and clinical data was approved by the institutional ethics committee of the First Affiliated Hospital with Nanjing Medical University (No. 2020-SR-130). And written informed consent was received from each patient. The records of 43 consecutive (January 2021–July 2021) patients with 76 benign breast tumors treated by FUS in our hospital were reviewed. Only 53 tumors of 28 patients were included in the radiomic analysis (Figure 1). These 28 patients were all female and the mean age was 27.53 years (range, 18–45 years). The inclusion criterion included the following: 1) US BI-RADS (Breast Imaging Recording and Data System) score 2–3 and mammography in addition for women older than 35 years with BI-RADS score ≤3; 2) benign breast disease proved by core-needle biopsy; 3) US image by the same senior radiologist before biopsy. The exclusion criteria were as follows: 1) abnormal echo around the lesion; 2) failed to complete the established treatment; 3) treated by different surgeons or different transducers; 4) the long diameter less than 5 mm.

### Ultrasound examination

All patients had undergone ultrasound examination before biopsy. The ultrasound examination was performed by the same senior radiologist using a real-time ultrasound system (DC-80S, Mindray, Shenzhen, China) with a 7.5 MHz linear array probe. The number of nodules and three orthogonal diameters (the largest diameter and two other perpendicular ones), distance to the skin, distance to the chest wall and the Alder grade was observed and recorded. The volume was calculated by the formula: V = πabc/6.

### Focused ultrasound surgery therapeutic procedure

An US-guided HIFU (USgHIFU) tumor therapeutic system (Mode-JC 200B, Chongqing Haifu Medical Technology Co. Ltd, Chongqing, China) was used to treat all patients. Therapeutic focused ultrasound energy was produced with an 18 cm-diameter transducer with a focal length of 8 cm, operated at a frequency of 1.0 MHz. The acoustic focus dimensions were 5 mm × 1.8 mm × 1.8 mm. The patient was positioned prone on the HIFU therapeutic system table with the skin overlaid to the lesion in contact with degassed water. The procedure was performed under local anesthesia. Dynamic real-time ultrasound imaging was used to observe the target lesion and the adjacent tissue, thus monitoring the HIFU ablation procedure. At the beginning, the coaxial US imaging device was used to establish the 3D image of the whole tumor. To establish a complete ablation plan, the whole tumor was divided into several slices of 3 mm separation. Sonication began from the deep to shallow of each slice. This process was repeated slice by slice to achieve complete tumor ablation. Once the gray scale covered the planned ablation area, the procedure was terminated. Technical parameters including treatment duration, sonication duration, mean power and sonication energy were recorded.

**FIGURE 2:**
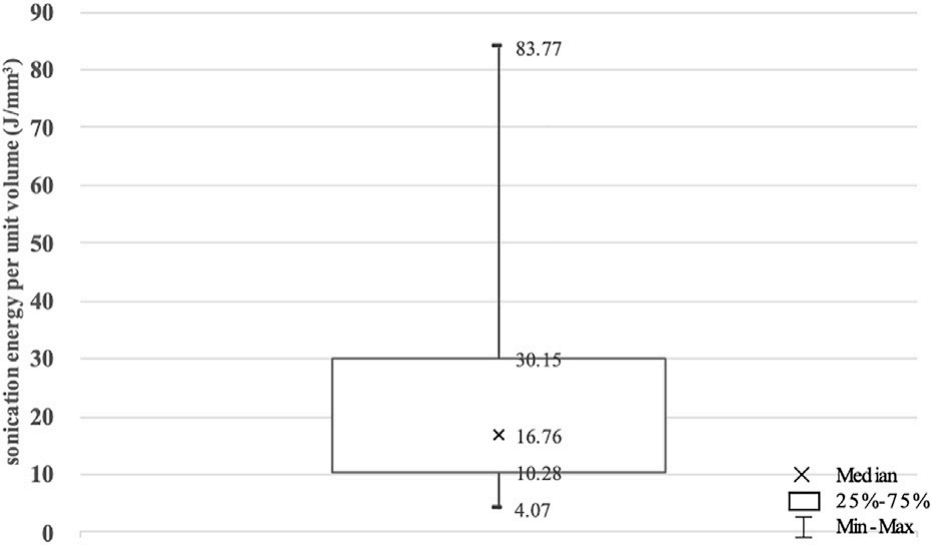
The sonication energy per unit volume for benign breast tumors enrolled in this study. Values are expressed as a median, interquartile range (IQR) and minimal and maximal values.

With the same strategy and protocol, a small number of tumors required far higher sonication energy than others, indicating harder to be ablated completely. The sonication energy per unit volume of each tumor was calculated to eliminate the effects of tumor size (Peng et al., 2015) (Figure 2). Three-quarter point was chosen as the cut-off to divide the 53 included tumors into high sonication energy (HSE, *n* = 14) and low sonication energy (LSE, *n* = 39) groups.

### Ultrasound feature extraction and radiomics analysis

**FIGURE 3:**
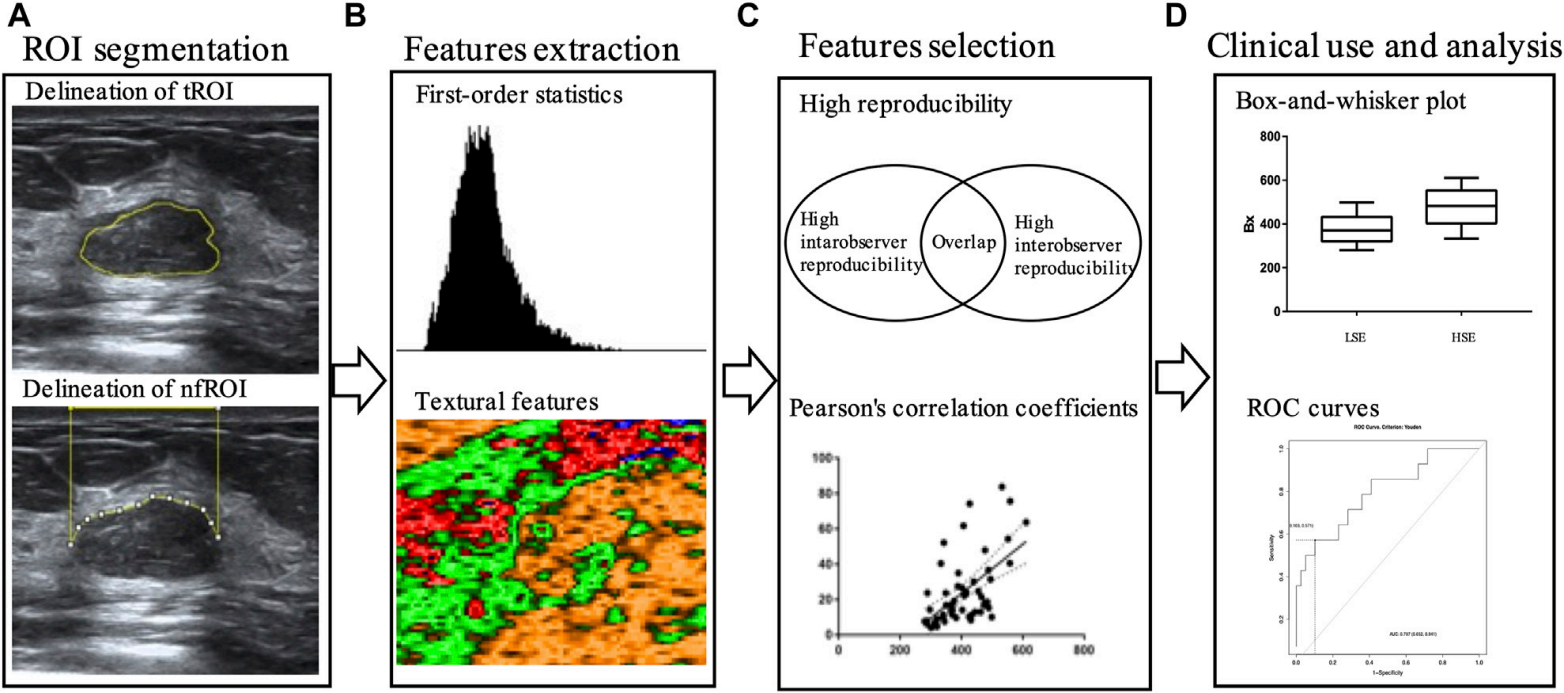
Workflow of main steps: **(A)** tROI and nfROI segmentation, **(B)** ultrasound radiomics features extraction with ImageJ, **(C)** features selection and **(D)** ROC analysis. tROI, region of interest of tumor; nfROI, region of interest of near field; LSE, low sonication energy; HSE, high sonication energy; ROC, receiver operator characteristic.

The workflow is illustrated in Figure 3. The ultrasound images were exported from our imaging system as DigitFal Imaging and Communications in Medicine format. Then, we used ImageJ to draw an outline of the region of interest (ROI) and extract the radiomics features. For each case, the tumor itself (tROI) and the near field tissue (nfROI) were delineated and analyzed separately. The ROI was delineated in the largest section of the tumor independently by two ultrasound radiologists with more than 5 years of experience. The tROIs was delineated closely along the inner edge of the tumor boundary, while the nfROIs was defined as the area from the skin to the shallow side of the tumor. 20 cases were chosen randomly to calculate the reproducibility of each radiomic feature using intra- and interobserver intraclass correlation coefficient (ICC). One radiologist repeated ROI segmentation twice in a week and the other delineated independently to calculate intra- and interobserver reproducibility, respectively.

### Statistical analysis

All statistical analyses were performed using R software (version 3.6.1, http://www.r-project.org). The continuous variables were shown as the mean ± standard deviation. Categorical and continuous variables were compared with χ2 test and Student’s t-test, respectively. Pearson correlation analysis was used for the correlation analysis. The diagnostic performance of the established models was evaluated by the ROC curve and area under the curve (AUC) value (Yang et al., 2020). A two-sided *p* value less than 0.05 was considered statistically significant.

## Results

### Baseline characteristics

A total of 53 tumors were included in this study. All tumors were ablated successfully as planned by USgFUS. The clinical characteristics of these tumors were summarized in Table 1. Except tumor size (length, *p* < 0.001 and volume, *p* = 0.02), there were no statistically significant differences in distance to the skin (*p* = 0.28), distance to the chest wall (*p* = 0.49), and Adler grade (*p* = 0.12) between group LSE and group HSE. The sonication energy per unit volume of group LSE and group HSE were 14.05 ± 6.44 J/mm^3^ and 52.03 ± 16.91 J/mm^3^ (*p* < 0.001), respectively.

**TABLE 1:** Baseline characters of benign breast tumors in Group LSE and Group HSE. Categorical variables are in n (%), and continuous variables are in mean ± SD. *p* values were calculated between Group LSE and Group HSE. LSE, low sonication energy; HSE, high sonication energy.

Parameter	LSE	HSE	*p* value
sonication energy per unit volume (J/mm^3^)	14.05 ± 6.44	52.03 ± 16.91	<0.001
Length (mm)	17.44 ± 6.29	9.92 ± 4.01	<0.001
Volume (mm^3^)	1999.64 ± 2,661.97	273.75 ± 283.35	0.02
distance to skin (mm)	6.26 ± 3.49	7.39 ± 2.81	0.28
distance to chest wall (mm)	13.28 ± 6.76	11.62 ± 9.78	0.49
Adler grade			0.12
0	8 (20.5)	6 (42.9)	
I	19 (48.7)	7 (50.0)	
II	12 (30.8)	1 (7.1)	

### Radiomics analysis

In total of 68 radiomics features were extracted from pre-ablation ultrasound images, of which 34 ultrasound features were extracted in tROI and 34 were in nfROI. 33 ultrasound radiomics features with high reproducibility (ICC >0.75) were selected for subsequent analysis.

### Correlation between ultrasound features and sonication energy and ROC curves

**FIGURE 4:**
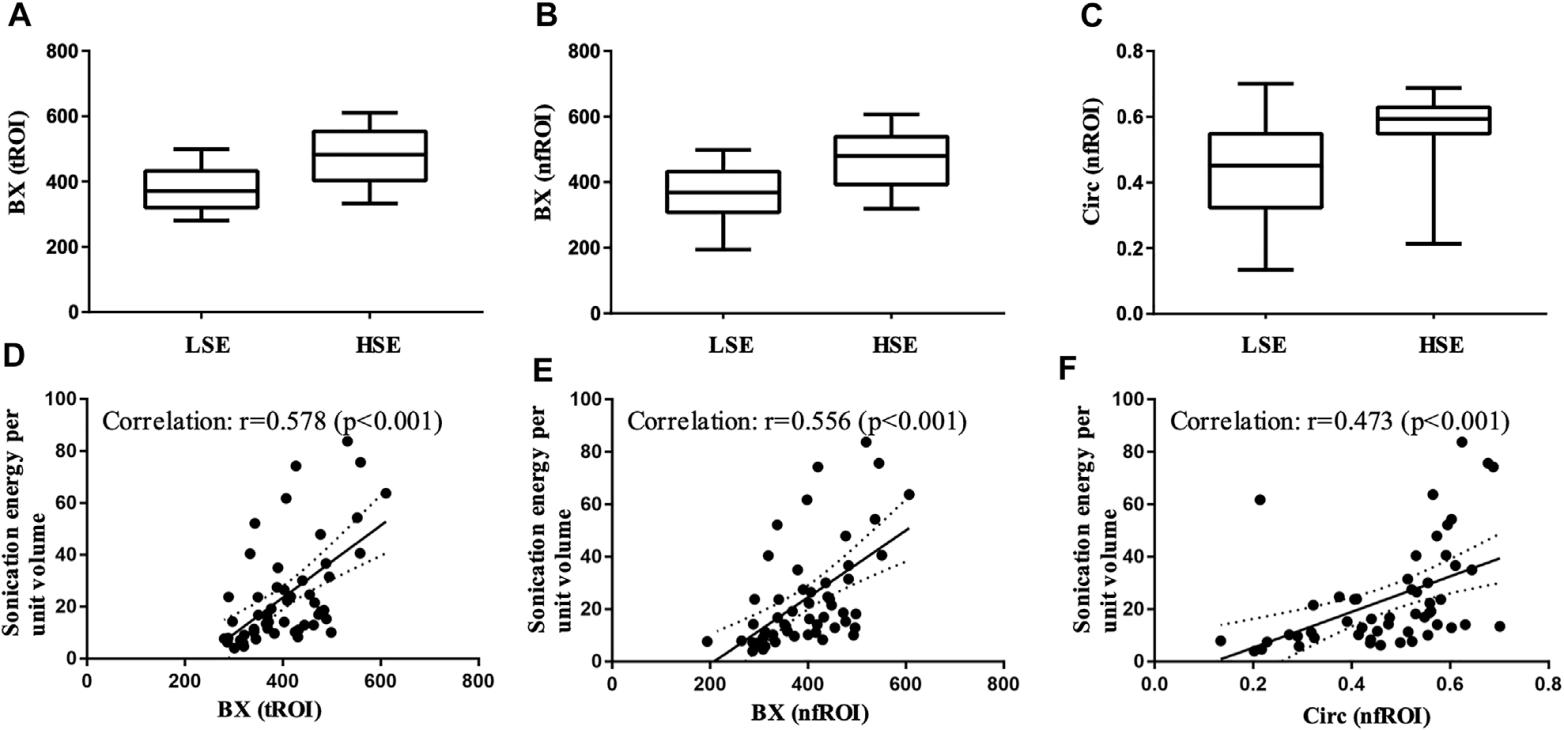
Box-and-whisker plot of **(A)** BX of tROI, **(B)** BX of nfROI and **(C)** Circ of nfROI in group LSE and group HSE. The correlation between **(D)** BX of tROI **(E)** BX of nfROI **(F)** Circ of nfROI and sonication energy per unit volume. Correlation was assessed using Pearson's correlation analysis. BX and Circ are radiomics features provided by ImageJ software. tROI, region of interest of tumor; nfROI, region of interest of near field; LSE, low sonication energy; HSE, high sonication energy.

**FIGURE 5:**
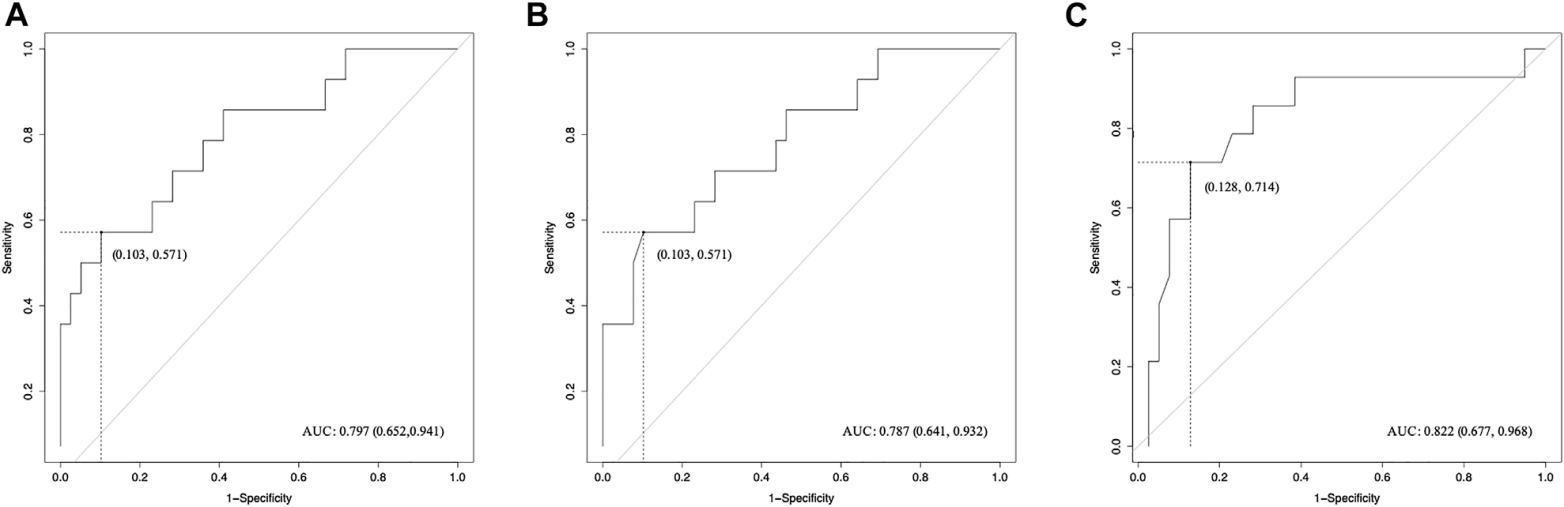
ROC curves of radiomics features for the prediction of sonication dose. **(A)** BX of tROI, **(B)** BX of nfROI and **(C)** Circ of nfROI predicting sonication dose. The cut-off values were estimated according to the maximum of the Youden index. tROI, region of interest of tumor; nfROI, region of interest of near field; ROC, receiver operator characteristic, AUC, the area under the ROC curve.

Pearson correlation analysis identified that 18 radiomics features of tROI and 13 features of nfROI were statistically significant between the two groups. Multiple linear regression analysis identified BX in tROI (*p* < 0.001), BX (*p* = 0.001) and Circ (*p* = 0.019) in nfROI were independently predictive features with sonication energy per unit volume (Figure 4). The results of Pearson correlation analysis and multiple linear regression analysis of ultrasound radiomics features in tROIs and nfROIs are provided in Supplementary Materials S1, S2, respectively. The results of ROC curve analysis of ultrasound image features and sonication energy were shown in Table 2. The AUCs of BX in tROI, BX and Circ in nfROI were 0.797 (95% CI: 0.65, 0.94), 0.787 (95% CI: 0.644 0.93), and 0.822 (95% CI: 0.68, 0.97), respectively (Figure 5).

**TABLE 2:** Diagnostic performance of BX (tROI), BX (nfROI) and Circ (nfROI) for the prediction of sonication energy. The cut-off values were determined at which the value of the Youden index was maximized. tROI, area of interest of tumor; nfROI, area of interest of near field; AUROC, area under the receiver operating characteristic curve; PPV, positive predictive value; NPV, negative predictive value; Positive/negative DLR, positive/negative diagnostic likelihood ratio.

	BX (tROI)	BX (nfROI)	Circ (nfROI)
AUC (95% CI)	0.797 (0.652, 0.941)	0.787 (0.641, 0.932)	0.822 (0.677, 0.968)
cut-off values	477	477	0.565
Sensitivity/specificity (%)	57/90	57/90	71/87
Correctly classified (%)	81	81	83
PPV/NPV (%)	67/85	67/85	67/89
Positive/negative DLR	5.57/0.48	5.57/0.48	5.57/0.33

## Discussion

In this study, we explored the differences of ultrasound image features between the minority of benign breast tumors which are ablated by high level of sonication energy and the majority of tumors which are ablated by low level of sonication energy. Although common clinical ultrasound characteristics, such as distance to skin, distance to chest wall, and Adler grade, were not statistically significant between the two groups. The correlation analysis and ROC curve indicated that 3 pre-ablation ultrasound image radiomics features could be considered as a novel index for the evaluation of the level of sonication energy needed by USgFUS.

Recently, FUS has been provided as a promising alternative to surgical procedures treating benign and malignant breast tumors. It is reported to be the only noninvasive and non-ionizing modality with potential benefits, such as less anesthesia involvement, low side effect, and good cosmesis effect (Hynynen et al., 2001; Kovatcheva et al., 2017; Imankulov et al., 2018; Peek et al., 2018). Without a needle or probe inserted into the target tumor, FUS transducer produces an ultrasound beam which passes through the overlying tissues and focuses on a target point. The affected region can be represented as a hyperechoic mark on the dynamic real-time ultrasound image as an indirect sign of thermal tissue damage. In general, reasonable ablation can be performed by controlling the three-dimensional motion of the focal point to cover the whole tumor in a regular way. However, in different cases, the exact sonication energy is individually adjusted based on the changes in greyscale on ultrasound image. It is reported in a clinical study that twice as much sonication energy was used in one patient (1/10) as the others (9/10) (Cavallo Marincola et al., 2015). In this study, significant high sonication energy was required by minority of benign breast tumors. It indicated that some particular types of benign breast tumors are difficult to be ablated. Characterizing these types is helpful to estimate treatment protocol and may become the key point to define the indications of USgFUS. In this study, we were working towards deep learning of pre-ablation images and providing three ultrasound radiomics features related to the therapeutic response of USgFUS.

*In vivo and in vitro* studies showed that volume of coagulative necrosis of FUS varied significantly in different tissues (Fry, 1993; Sibille et al., 1993; Wang et al., 2003). Wang *et al.* proposed the concept of BFR, defined as individual coagulative necroses induced by a single exposure of FUS to draw attention to the influence of tissue structure and its functional status (Wang et al., 2003). On one hand, the acoustic environment differs from kinds of tissues. On the other hand, during the process of FUS, the acoustic environment changes dynamically along with microbubbles and necrosis produced in tissue (Sibille et al., 1993; Rabkin et al., 2006). Microbubbles could interfere with sound passage, leading to the lesion extension toward a sound source. At the interface between necrosis tissue and normal tissue, reflection, refraction, scatter and diffraction of ultrasound could be amplified, accelerating tissue ablation in the rest (Fry, 1993). These could explain partially why bigger volume required lower level of sonication energy per unit volume.

Tissues in the near field will absorb, reflect, and scatter ultrasonic waves and consequently result in ultrasonic energy attenuation. Studies have shown that the ultrasonic energy required to ablate the same unit volume of target tissue was significantly related to focus depth and nature of tissue (Johnston and Dunn, 1976; Li et al., 2006). In this study, although the distance from tumor to skin between two groups showed no significant differences, radiomics analysis of near filed did provide two ultrasound image features which played an independent predictive role on therapeutic energy of FUS. This finding justified that nature of near field tissue had effects on energy delivery.

For years, studies were conducted to explore the factors on sonication dose, in hope of optimizing the indications and improving the effectiveness of FUS. Johnson *et al.* analyzed the relationship between the lesion volumes produced in cat brain and the energy absorbed per unit volume of the lesion (Johnston and Dunn, 1976). Peng et al. analyzed factors affecting the amount of energy required for tissue ablation per unit volume and built a dosimetry model of high-intensity focused ultrasound ablation for uterine fibroids (Peng et al., 2015). However, the acoustic characteristic of the ablated tissue was difficult to be described quantitatively. Then, radiomics made it possible. Li et al. built a T2 MR-based radiomics prediction model incorporating radiomics features and clinical parameters to predict the response to FUS in patients with adenomyosis (Li et al., 2021). The findings of our study showed that radiomics analysis of pre-ablation ultrasound images could provide clues to therapeutic response to USgFUS in patients with benign breast tumor. It indicates that ultrasound can not only guide and treat benign breast tumors, but also predict the sonication dose.

There are several limitations in this study. First of all, radiomics features were only extracted from greyscale ultrasonography images. Additional data from color Doppler ultrasonography, ultrasound elastography, and contrast-enhanced ultrasound may improve performance of the radiomics. Secondly, the present study was a retrospective study. And few clinical parameters were involved in this analysis. With follow-up data, further radiomics analysis will provide more valuable findings. In addition, the sample size of the study was small. In the future, large, multi-center clinical studies are necessary to further validate the findings of this study.

## Conclusion

We found that differences in pre-ablation ultrasound radiomics features were related to the level of sonication energy needed by USgFUS. Ultrasound radiomics analysis can predict benign breast tumors suitable to be ablated by FUS with low energy. More importantly, it would exclude cases requiring an extremely high doses of energy which may cause long process, severe pain, and peripheral tissue injury. It could be a key role for clinical indications of breast FUS. Still, prospective clinical trials with large sample size are needed to confirm the predictive effect of these radiomics features. Furthermore, it would be of great significance to develop a prediction model based on radiomics features and clinical parameters to predict the sonication energy in patients with benign breast tumors.

## Data Availability

The raw data supporting the conclusions of this article will be made available by the authors, without undue reservation.
