# Supplementary material for: Ultrasound radiomics features predicting the dosimetry for focused ultrasound surgery of benign breast tumor: A retrospective study
Source: Front Genet. 2022 Sep 6;13:969409. doi: 10.3389/fgene.2022.969409 (PMC9479455; doi:10.3389/fgene.2022.969409)
Supplement: Supplementary file 1 [file Table1.DOCX]

|  | **Pearson correlation analysis** | | **Multiple linear regression** | |
| --- | --- | --- | --- | --- |
| **Variables** | ***r^2^* value** | ***P* value** | ***b* coefficient** | ***P* value** |
| **Area** | 0.146 | 0.005 | NA | 0.51 |
| **X** | 0.097 | 0.023 | NA | 0.354 |
| **XM** | 0.085 | 0.034 | NA | 0.427 |
| **Perim** | 0.177 | 0.002 | NA | 0.467 |
| **BX** | 0.334 | <0.001 | 0.139 | <0.001 |
| **BY** | 0.084 | 0.036 | NA | 0.05 |
| **Width** | 0.173 | 0.002 | NA | 0.519 |
| **Height** | 0.149 | 0.004 | NA | 0.515 |
| **Major** | 0.181 | 0.001 | NA | 0.5 |
| **Minor** | 0.160 | 0.003 | NA | 0.391 |
| **Angle** | 0.127 | 0.009 | NA | 0.053 |
| **Feret** | 0.171 | 0.002 | NA | 0.54 |
| **IntDen** | 0.090 | 0.029 | NA | 0.724 |
| **RawintDen** | 0.090 | 0.029 | NA | 0.724 |
| **FeretX** | 0.331 | <0.001 | NA | 0.914 |
| **FeretAngle** | 0.085 | 0.034 | NA | 0.107 |
| **MinFeret** | 0.156 | 0.003 | NA | 0.426 |
| **Solidity** | 0.099 | 0.022 | NA | 0.109 |

**Supplementary material 1** Ultrasound radiomics features in tROIs related to sonication dose. The definition of these radiomics features belongs to ImageJ software. tROI, region of interest of tumor.

|  | **Pearson correlation analysis** | | **Multiple linear regression** | |
| --- | --- | --- | --- | --- |
| **Variables** | **r^2^ value** | **P value** | **b coefficient** | **P value** |
| **X** | 0.094 | 0.026 | NA | 0.598 |
| **XM** | 0.092 | 0.027 | NA | 0.621 |
| **YM** | 0.075 | 0.047 | NA | 0.395 |
| **Perim** | 0.100 | 0.021 | NA | 0.489 |
| **BX** | 0.309 | <0.001 | 0.1 | 0.001 |
| **Width** | 0.179 | 0.002 | NA | 0.440 |
| **Major** | 0.158 | 0.003 | NA | 0.607 |
| **Circ** | 0.224 | <0.001 | 42.457 | 0.019 |
| **Feret** | 0.099 | 0.021 | NA | 0.320 |
| **FeretX** | 0.309 | <0.001 | NA | 0.088 |
| **AR** | 0.147 | 0.004 | NA | 0.130 |
| **Round** | 0.243 | <0.001 | NA | 0.420 |
| **Solidity** | 0.206 | 0.001 | NA | 0.749 |

**Supplementary material 2** Ultrasound radiomics features in nfROIs related to sonication dose. The definition of these radiomics features belongs to ImageJ software. nfROI, region of interest of near field.
